# Cross-cultural adaptation of the stroke action test for Italian- speaking people

**DOI:** 10.1186/s12883-015-0335-z

**Published:** 2015-05-10

**Authors:** Licia Denti, Barbara Marcomini, Silvia Riva, Peter J. Schulz, Caterina Caminiti, for EROI (Educazione e Ritardo di Ospedalizzazione per Ictus) study group

**Affiliations:** Geriatric Clinic University Hospital of Parma, Via Gramsci 14, 43100 Parma, Italy; Research and Innovation Unit, University Hospital of Parma, Via Gramsci 14, 43100 Parma, Italy; Institute of Communication and Health, Università della Svizzera Italiana, Via G. Buffi 6 CH, 6900 Lugano, Switzerland

**Keywords:** Stroke awareness, STAT, Knowledge, Cross cultural adaptation

## Abstract

**Background:**

Assessing the level of public stroke awareness is a prerequisite for development of community educational campaigns aimed at reducing prehospital delay of stroke patients. The Stroke Action Test (STAT) is a validated instrument specifically developed in the United States with the objective to assess the public’s readiness to respond to stroke. Our purpose was to perform the cross-cultural adaptation of the original version of STAT to be applied to the Italian population.

**Methods:**

The process of cross-cultural adaptation has been performed according to guidelines, intended for questionnaires of self-report health status measures, following five steps: forward translation, synthesis, back translation, approval by an Expert Committee and test of the pre-final version. For this last step, 31 adults were asked to rate each item in terms of adequacy of content, clarity of wording and usefulness, according to a 3-point scale. The final version has been administered to a sample of 202 volunteers to assess its acceptability and reliability in terms of the internal consistency.

**Results:**

The pre-final version of the STAT was developed taking into accounts few and minimal discrepancies between the two back translations and the original version of the instrument. Most items were judged as adequate, easy to understand and useful, according to the frequency of high scores (>50 %) given by the adaptation sample. As for further testing of the adapted final version, completeness of item response was very good. Distribution of scores ranged from 0 to 100 %, without any floor or ceiling effect, with a percentage of the lowest scoring of 1.5 % for the 28-item test and 2.5 % for the 21-item test and a percentage of the highest scoring of 1 % for both tests. Internal consistency was high for both the 28-item and 21-item tests (Cronbach alpha = 0.85 and 0.84, respectively).

**Conclusions:**

The process used to perform the cross-cultural adaptation of the questionnaire was successful. The Italian version of STAT demonstrated good acceptability and psychometric properties and is now available to assess stroke awareness in Italian people.

## Background

Stroke awareness can be defined as the capacity of early recognition of stroke symptoms by patients and/or witnesses, along with their knowledge of the most appropriate action to take in response to stroke onset, that is, quick referral to Emergency Medical Services.

The public levels of awareness of stroke warning signs and risk factors have been reported to be relatively low in several contexts, notably in higher risk, older age groups [1, 2].

This issue is quite relevant, considering that stroke represents a major clinical burden in Italy, as well as in other high-income countries, with an annual incidence rate standardized to the Italian population of at least 175/1.000.000 in men and 130/1.000.000 in women, with some differences across regions [3, 4]. Besides, the incidence is expected to rise in the next few years due to the ageing population, so that an increasing amount of people are expected to seek help for stroke symptoms.

Therefore, increasing stroke awareness through public educational campaigns has been widely advocated as a means to reduce pre-hospital delay and increase the potential for patient access to proven therapies, such as intravenous tissue plasminogen activator (tPA) [5].

For developing effective educational interventions, as well as for evaluating and monitoring their effectiveness, a validated tool for a preliminary assessment of the extent of stroke awareness in the community is needed.

According to several reports [1, 2, 4–10], stroke symptom knowledge and the intention to call the Emergency service are not associated. It should be noted that the American Heart Association advises that calling 911 should be the first and only response to suspected stroke symptoms, because the use of emergency medical services (EMS) is associated with earlier presentation to the hospital and greater rate of recombinant tPA use [5, 11–13].

In this view, it is important to assess not only the respondent’s theoretical knowledge of stroke warning signs, but also his/her ability to connect symptoms with appropriate actions.

To our knowledge, two standardized questionnaires are available, which include questions about both stroke knowledge and the proper response to stroke. The Stroke Awareness Questionnaire (SAQ) [11] was developed from surveys from Ireland [14, 15], Europe [16], the United States [17–19] and Australia [20]. Although the authors declared that the instrument was tested with stroke- related professionals and members of the public, no formal validation study has been published.

The Stroke Action Test (STAT) is a validated instrument specifically developed in the United States with the objective to assess the public’s readiness to respond to stroke [21]. In the development and validation paper, the authors declare that STAT “directly assesses a critical aspect of practical stroke knowledge that has been largely overlooked by other published tests for the assessment of stroke warning sign knowledge.” In fact, it contains items that require the respondent to associate individual symptoms with the most appropriate action. The reliability (Cronbach alpha 0.83) and validity of STAT scores have been reported as good, according to a preliminary test on a sample of 249 subjects from community-based organizations [21].

Few reports are available on the use of STAT in non-English-speaking countries. In particular, it has been employed in a nationwide survey in the Czech Republic [22, 23] after being translated and tested on 20 volunteers for clarity and comprehension. However, as a general rule, to maintain the validity of the original instrument while taking into consideration cultural differences, the mere linguistic translation is not sufficient. One must refer to specific guidelines for the process of cross-cultural adaptation of self-report measures [24–26], to ensure equivalence between the original and target versions of the questionnaire.

Hence, the purpose of this study was to adapt the original version of STAT to be applied to an Italian population, and to evaluate its acceptability and internal consistency in a sample of Italian respondents. This study was sponsored by the Emilia Romagna Region, as part of a broader project aiming to evaluate the effectiveness of an educational campaign in reducing access times to hospital of patients with stroke (Educazione e Ritardo di Ospedalizzazione per Ictus, EROI project, trial registration http://clinicaltrials.gov NCT01881152). This study was approved by Parma Provincial Ethics Committee in February 2012.

## Methods

### The instrument

The STAT is a 28-item written instrument that measures the potential response of a person to stroke, by his or her ability to associate specific symptoms with the most appropriate action [19]. STAT items include 21 stroke symptoms representing all 5 groups of warning signs, as well as 7 non-stroke symptoms. Eleven items involve sudden unilateral numbness or weakness of the face, arm or leg, or trouble speaking or understanding. Two items contain a common stroke syndrome (for example sudden right-side weakness of the face and arm, together with trouble speaking). The 7 non-stroke symptoms represent both urgent and non-urgent medical conditions.

For each item, the respondent is required to answer the question, “If this happened to you or an adult friend/relative, what would you do?” by selecting 1 of 4 response options: (1) call 911 immediately; (2) call doctor’s office immediately; (3) wait 1 h and then decide; or (4) wait 1 day and then decide. For scoring purposes, each correct response receives 1 point; incorrect responses receive 0 points. The total score is reported as the percentage of correct responses.

### Adaptation process

The original version of the STAT was cross culturally adapted to Italian following the five steps described by Guillemin et al. [24] and Beaton et al. [25], intended for questionnaires of self-report health status measures.

Step 1 - Forward translation: The questionnaire was translated into Italian by two certified translators with Italian as their mother tongue. One had a clinical background, while the other one was not aware of the concept being quantified. In this way, two Italian versions of the questionnaire, labeled T1 and T2, were produced.

Step 2- synthesis: The two translators met to discuss their work and agreed on a common Italian version (T12). Discrepancies between the two versions were identified and discussed, and were resolved by consensus between the translators.

Step 3- back translation: two certified translators (native speakers of English), who were unaware of the concept of the questionnaire and had no medical background, independently translated T12 into English, thus producing two back translations of the questionnaire. This step of the process was useful to identify any important semantic or conceptual differences between the English and Italian versions. In this stage, the back translators were asked to highlight any difficulties or uncertainties with regard to wording and diction.

Step 4- Expert Committee: a Committee was set up, composed of the four translators, health professionals (two stroke experts, a neurologist and a geriatrician), one methodologist-biostatistician and one linguist. The back translations were compared with the original version in terms of semantic, idiomatic, experiential and contextual equivalence, to identify discrepancies. A consensus was reached and the pre-final version of the questionnaire was obtained.

Step 5- test of the pre-final version: the pre-final version was submitted to a sample of volunteers, recruited from the Italian voluntary Stroke organization ALICe (Associazione per la Lotta all’Ictus Cerebrale) during World Stroke Day on October 29^th^ 2012 in Parma. They were requested to rate each question in terms of adequacy of content (if the wording and content of the question was adequate to the context), clarity of wording, and usefulness, using 3-point rating scales, as well as to provide comments and suggestions.

### Further testing of the adapted version

After the translation and adaptation process, the literature highly recommends additional testing to ensure that the new version has the measurement properties needed for the intended application [25]. For this purpose, we used data from a sample of 202 volunteers of four provinces of Northern Italy (Parma, Piacenza, Reggio Emilia and Modena) collected for planning the educational campaign in the broader EROI project. A minimum sample size of 199 participants was estimated, according to the number of the questionnaire items (28 items), a probability of Type 1 error 0.05 and a power 0.80 [27].

Criteria for inclusion were age over 19, self-assessed ability to read Italian, and lack of professional medical training. The respondents were contacted by members of the organization ALICe at public events for fundraising, in malls and gyms. To ensure that our sample was really representative of our population, we created a demographic table that contained the desired number of respondents for each Province, in each of the categories of geographical area (urban, rural, mountain), age, sex and race,, in accordance with local demographics. Once someone was contacted, questions were first asked to determine if he/she matched an unfilled demographic. Then, only those respondents whose demographic characteristics matched an unfilled category were interviewed.

### Ethics approval

The study was carried out according to the Helsinki Declaration and was approved by the Parma Provincial Ethics Committee. All participants gave written, informed consent.

### Statistical analysis

As for the test on the pre-final version, descriptive statistics were used, considering the scores attributed to each item of the STAT in relation to the three aspects we considered. To facilitate comprehension, and highlight differences, frequencies were displayed by means of histograms and 50 % cut offs were considered as significant for analysis by the Committee, being already used in previous reports [28].

As for further testing of the adapted version, the statistical analysis was planned in accordance with the statistical protocol used for validation of the original STAT [21]. Descriptive statistics were used to summarize the characteristics of subjects, mean item and test scores, and frequencies with which they chose each response option.

As measures of the acceptability of the final version, completeness of item response, distribution of the scores, and ceiling and floor effects (patients scores at either extreme of the scale) were analyzed using descriptive statistics.

To determine the reliability of the questionnaire in terms of internal consistency, the Cronbach’s alpha coefficient was used. A “high” value of alpha is often used as evidence that the items measure an underlying (or latent) construct [29]. For each item, this test shows how alpha would vary if that particular items were deleted. If the item has a strong relationship with the entire STAT, alpha will turn out to be very small. In contrast, if the Cronbach’s alpha remains high, it would indicate that the item poorly contributed to the STAT’s internal consistency.

All data analyses were performed using the SPSS PASW Statistics software (version 18.0).

## Results

### Adaptation

Concerning Stage II and III of the adaptation process, few and minimal discrepancies between the two back translations and the original version of the instrument were noted, indicating that T12 (synthesis of the 2 forward translations) was substantially accurate.

In particular, a few English words proved problematic, having multiple translations into Italian, such as “dizziness”, “trouble”, “confusion” and “numbness”. The final decision was in favor of the term that allowed for the best balance between medical and informal wording. In all cases, the final translation was proved to be correct, according to the back-translation.

From a stylistic point of view, it was decided to keep the language informal and simple, following the English version, although in some cases the wording was slightly modified to better suit the Italian style.

Thirty one adults participated in the evaluation of the items, completing Stage V of the cross-cultural adaptation process (Table 1).

**Table 1:** Characteristics of two samples enrolled for STAT adaptation and for testing the adapted version

Variable	Adaptation sample 31	Testing sample (202)
Sex % (n)		
Female	42 (13)	55 (111)
Male	58 (18)	45 (92)
Age		
Mean (SD)	64.3 (10.9)	51.5 (16.8)
Range	41–85	19–89
Education (highest level achieved) % (n)		
Primary school	6.5 (2)	15.1 (31)
Secondary school	9.7 (3)	23.3 (45)
High school	54.8 (17)	47 (95)
University graduate	29.0 (9)	
	14.4 (29)	

**Fig. 1:**
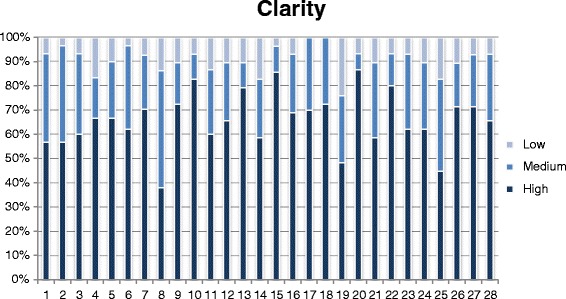
People ratings on clarity of wording of each question, expressed on a 3-point Likert scale

**Fig. 2:**
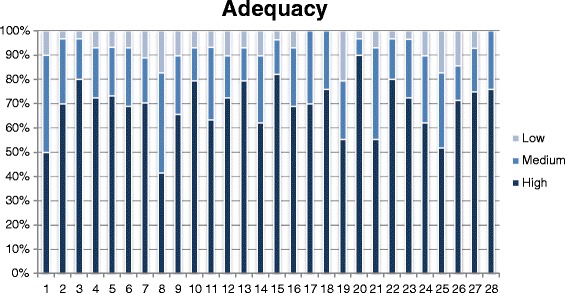
People ratings on adequacy of content of each question, expressed on a 3-point Likert scale

**Fig. 3:**
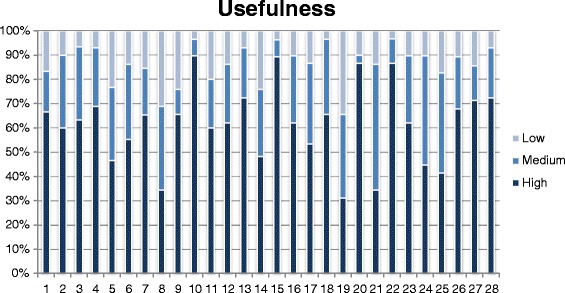
People ratings on usefulness of each question, expressed on a 3-point Likert scale

Subject ratings of each item concerning the three dimensions of adequacy of content, clarity of wording and usefulness expressed on a 3-point scale, are depicted in Figs. 1, 2, and 3, respectively. For each dimension, a histogram was constructed, with the 28 questions as categories on the X-axis and the frequency of the 3 scores, low, medium and high, expressed as percentages, on the Y-axis.

For most questions the frequency of high scores was > 50 % for adequacy of content and clarity of wording, while usefulness of several questions was most frequently rated low-medium.

These findings were discussed by the Committee. Table 2 features the questions that required discussion and revision, with the correspondent explicative notes.

**Table 2:** The questions that required discussion and revision by the Committee

Item	Notes
8. My gym partner complained about his right hand hurting and feeling numb while he was lifting weights. He was able to finish his workout anyway.	Clarity, adequacy and usefulness rated as low. Unusual scenario for Italian context: physical activity is usually carried out individually, often not in gyms, and weight lifting is not commonly practiced. The question was rephrased and retained, because it was agreed that it could facilitate the applicability of the questionnaire to younger people, thus increasing its generalizability.
25. Sudden weakness of the face especially on one side.	Clarity and usefulness rated as low. The question was rephrased and retained.
5. Sudden numbness of the leg, especially on one side.	Rated by most subjects as of low-medium usefulness. The questions were retained, as this judgment was likely influenced by the level of knowledge of stroke, which is what the questionnaire intended to measure
14. Sudden weakness of the arm especially on one side.	
21. Sudden severe headache with no known cause.	
24. I noticed that he kept covering and uncovering his eyes and blinking. He told me, “I can’t see.” A few minutes later everything was fine again	

The notes describe the Committee’s final decisions

All questions were maintained in the final version and two of them were properly rephrased to increase clarity. As for their low usefulness score, the group considered that the judgment of usefulness was likely influenced by the level of knowledge of stroke, which is what the questionnaire intended to analyze.

Overall, we found some associations between scoring rates in the three domains: questions which scored low for clarity, also scored low for adequacy and usefulness, which suggests strong interaction of the three types of evaluation and the overall influence of the level of stroke knowledge.

### Further testing of the adapted version

Table 2 shows the demographic characteristics of the 202 lay people enrolled for testing the adapted version of the questionnaire.

The mean overall STAT score (based on all 28 items) was 52.5 % (SD 16.6). The mean score on the 21 items containing stroke symptoms was 44.5 % (SD 21.8).

This means that on average, participants correctly chose to call 991 in 44.5 % of the situations with stroke symptoms. Furthermore, 44.1 % of the respondents achieved a 21-item score ≥ 50, which was considered as “adequate” in previous reports [22, 23].

Completeness of item response was very good. Only 38 missing answers were identified, accounting for a missing data percentage of 0.67 %, which indicates that the questionnaire had good acceptability.

Distribution of scores ranged from 0 to 100 %, without any floor or ceiling effect, with a percentage of the lowest scoring of 1.5 % for the 28-item test and 2.5 % for the 21-item test and a percentage of the highest scoring of 1 % for both tests.

Internal consistency was high for both the 28-item and 21-item tests (Cronbach alpha = 0.85 and 0.84, respectively).

Reliability analysis showed that only one item, not related to stroke (“His finger joints were sore, and then a finger locked-up so he couldn’t open his hand”) poorly contributed to the reliability of the questionnaire as the Cronbach’s alpha value increased after deleting it, while all the other items highly contributed to the reliability of STAT as the Cronbach’s alpha value decreased after deleting them.

## Discussion

The purpose of this study was to translate and culturally adapt the Italian version of the STAT questionnaire, following a systematic standardized process, which can ensure the semantic and conceptual equivalence of the translated version to the original tool.

The process of forward and back translation for the development of the pre-final version of the questionnaire was carried out without major difficulties. As for the few discrepancies in terms of wording and style, an agreement between the translators was easily found.

No major problems were identified in the evaluation phase. Clarity of wording and adequacy of content were rated as high by at least 50 % of respondents for 26 items, while the rating of usefulness was the highest for 21 of these.

The content validity of the final version was supported by good completeness of item response, adequate score distribution, and absence of floor and ceiling effect.

The internal consistency of the adapted questionnaire turned out as similar to that reported for the original version, according to the high value of Cronbach coefficient alpha (0.85 for the 28-item test). Only for 1 question, not regarding stroke-related symptoms, the contribution to the questionnaire’s internal consistency turned out as low.

The method we employed to perform the cross-cultural adaptation of STAT deserves some further considerations. It is generally agreed that a questionnaire that has been developed in a specific cultural context can be used for another culture only if there is equivalence between the original and adapted questionnaires [30]. To this aim, several methods are available and most of them include use of committees, focus groups, and back translations. According to a recent review [31], evidence for the best methods is lacking, and the authors conclude that most of the available methods would achieve comparable results, and choosing one is a matter of preference and logistic.

We chose the strategy developed by Beaton and collaborators, which includes forward and back-translations, as it is one of the most frequently employed and because we had previous experience with it. So we cannot exclude that other methods might have been more appropriate for achieving our primary objective, which can be considered one major limitation of our study.

The main strength of this study is that it has made available, for the first time, a standardized and validated questionnaire for the assessment of stroke awareness in the Italian population. This tool may be used, for instance, to assess stroke awareness in our community in relation with pre-hospital delay. Also, the choice to adapt a tool already validated in another language, instead of devising a new questionnaire, will enable to perform cross-cultural research, providing a reliable instrument to compare the results of studies on stroke knowledge in our community with those reported for other populations.

Actually, we intend to use the translated questionnaire for the preliminary context analysis that is part of a project aimed at developing a public educational campaign to reduce prehospital delay of stroke patients. A preliminary analysis of the level of stroke awareness in our community and of the clinical scenarios that are wrongly perceived as non-urgent by people can help in defining the content of the educational message, which can be designed in accordance with the real educational needs within the target community. It is well known that any educational intervention should be developed after verifying the validity of pre-specified theoretical assumptions within the local cultural context [32, 33]. The preliminary context analysis, which is also aimed to assess the determinants of prehospital delay of stroke patients in the four participating provinces, is one of the three phases of EROI project, along with the development and evaluation of the educational campaign.

In addition, the instrument can be useful to assess the effectiveness of educational interventions on stroke awareness, as previously reported [23]. The scarce impact of the campaign conducted throughout the Czech Republic was demonstrated using as measure of the primary outcome a STAT score ≥ 50 %.

In conclusion, the results of this study confirmed that the process used to perform the cross-cultural adaptation of the questionnaire was successful. The Italian version of STAT demonstrated good acceptability and psychometric properties, and our results were comparable to those obtained with the original version of the questionnaire. The Italian STAT questionnaire is thus available to be used by researchers to measure stroke awareness in Italian people and make comparisons with data from other countries. Furthermore, a better understanding of people’s beliefs about stroke, in addition to symptom recognition, will allow the development of public educational campaigns to increase stroke awareness and reduce pre-hospital delay.

## Abbreviations

STAT: Stroke Awareness Test
ALICe: Associazione Lotta all’Ictus Cerebrale
EROI: Educazione e Ritardo di Ospedalizzazione per Ictus

## References

[1] Nicol M, Thrift AG (2005). Knowledge of risk factors and warning signs of stroke. Vascular Health Risk Manag.

[2] Jones SP, Jenkinson AJ, Leathley MJ, Watkins CL (2010). Stroke knowledge and awareness: an integrative review of the evidence. Age Ageing.

[3] Quaderni del Ministero della Salute. N 2. Marzo-aprile 2010. Organizzazione dell’assistenza all’ictus: le stroke units. www.quadernidellasalute.it

[4] Sacco S, Stracci F, Cerone D, Ricci S, Carolei A (2011). Epidemiology of stroke in Italy. Int J Stroke.

[5] Moser DK, Kimble LP, Alberts MJ, Alonzo A, Croft JB, Dracup K, Evenson KR, Go AS, Hand MM, Kothari RU, Mensah GA, Morris DL, Pancioli AM, Riegel B, Zerwic JJ. Reducing Delay in Seeking Treatment by Patients with Acute Coronary Syndrome and Stroke. A Scientific Statement From the American Heart Association Council on Cardiovascular Nursing and Stroke Council 10.1097/01.JCN.0000278963.28619.4a 17589286

[6] Fussman C, Rafferty AP, Lyon_callo S, Morgenstern LB, Reeves MJ (2010). Lack of association between stroke symptoms knowledge and intent to call 911: a population-based survey. Stroke.

[7] Williams LS, Bruno A, Rouch D, Marriott DJ (1997). Stroke patients’ knowledge of stroke. Influence on time to presentation. Stroke.

[8] Ritter MA, Brach S, Rogalewski A, Dittrich R, Dziewas R, Weltermann B (2007). Discrepancy between theoretical knowledge and real action in acute stroke: self-assessment as an important predictor of time to admission. Neurol Res.

[9] Barr J, McKinley S, O’Brien E, Herkes G (2006). Patient recognition of and response to symptoms of TIA or stroke. Neuroepidemiology.

[10] Ellis C, Egede LE (2009). Stroke recognition among individuals with stroke risk factors. Am J Med Sci.

[11] Schroeder EB, Rosamond WD, Morris DL, Evenson KR, Hinn AR (2000). Determinants of use of emergency medical services in a population with stroke symptoms: the second Delay in Accessing Stroke Healthcare (DASH II) study. Stroke.

[12] Morris DL, Rosamond W, Madden K, Schultz C, Hamilton S (2000). Prehospital and emergency department delays after acute stroke: the Genentech stroke presentation survey. Stroke.

[13] Hickey A, Holly D, McGee H, Conroy R, Shelley E (2012). Knowledge of stroke risk factors and warning signs in Ireland: development and application of the stroke awareness questionnaire (SAQ). Int J Stroke.

[14] Morgan K, McGee H, Watson D et al. SLAN 2007: Survey of Lifestyle, Attitudes & Nutrition in Ireland. Main Report 2008

[15] Parahoo K, Thompson K, Cooper M, Stringer M, Ennis E, McCollam P (2003). Stroke: awareness of the signs, symptoms and risk factors – a population-based survey. Cerebrovasc Dis.

[16] Rossnagel K, Jungehulsing GJ, Nolte CH (2004). Out-of-hospital delays in patients with acute stroke. Ann Emerg Med.

[17] Pancioli AM, Broderick J, Kothari R (1998). Public perception of stroke warning signs and knowledge of potential risk factors. JAMA.

[18] Blades LL, Oser CS, Dietrich DW (2005). Rural community knowledge of stroke warning signs and risk factors. Prev Chronic Dis.

[19] Rowe AK, Frankel MR, Sanders KA. Stroke awareness among Georgia adults: epidemiology and considerations regarding measurement 11440330

[20] Yoon SS, Heller RF, Levi C, Wiggers J, Fitzgerald PE (2001). Knowledge of stroke risk factors, warning symptoms, and treatment among an Australian urban population. Stroke.

[21] Billings-Gagliardi S, Mazor KM (2005). Development and validation of the stroke action test. Stroke.

[22] Mikulik R, Bunt L, Hrdlicka D, Dusek L, Vacklavik D, Kryza J (2008). Calling 911 in response to stroke. A nationwide study assessing definitive individual behavior. Stroke.

[23] Mikulik R, Goldemund D, Reif M, Brichta J, Neumann J, Jarkovsky J (2011). Calling 991 in response to stroke: non change following a four-year educational campaign. Cerebrovasc Dis.

[24] Guillemin F, Bombardier C, Beaton D (1993). Cross-cultural adaptation of health related quality of life measures: literature review and proposed guidelines. J Clin Epidemiol.

[25] Beaton DE, Bombardier C, Guillemin F, Ferraz MB (2000). Guidelines for the process of cross-cultural adaptation of self-report measures. Spine (PhilaPa 1976).

[26] Wild D, Grove A, Martin M, Eremenco S, McElroy S, Verjee-Lorenz A (2005). ISPOR task force for translation and cultural adaptation: principles of good practice for the translation and cultural adaptation process for patient-reported outcomes (PRO) measures: report of the ISPOR task force for translation and cultural adaptation. Value Health.

[27] Bonett DG (2002). Sample size requirements for testing and estimating coefficient alpha. J Educ Behav Stat.

[28] Caminiti C, Diodati F, Filiberti S, Marcomini B, Annunziata MA, Ollari M (2010). Cross-cultural adaptation and patients’ judgments of a question promps list for Italian-speaking cancer patients. BMC Health Serv Res.

[29] Tavakol M, Dennick R (2011). Making sense of Cronbach’s alpha. Int J Med Ed.

[30] Gjersing L, Caplehorn JRM, Clausen T (2010). Cross-cultural adaptation of research instruments: language, setting, time and statistical considerations. BMC Met Res Methodol.

[31] Epstein J, Santo RM, Guillemin F (2015). A review of guidelines for cross-cultural adaptation of questionnaires could not bring out a consensus. J Clin Epidem.

[32] Bartholomew LK, Parcel GS, Kok G (1998). Intervention mapping: a process for designing theory- and evidence-based health education programs. Health Educ Behavior.

[33] Morgenstern LB, Staub L, Chan W, Wein TH, Bartholomew LK, King M (2002). Improving delivery of acute stroke therapy: the TLL temple foundation stroke project. Stroke.

